# Overexpression of peanut (*Arachis hypogaea* L.) *AhGRFi* gene enhanced root growth inhibition under exogenous NAA treatment in *Arabidopsis thaliana*

**DOI:** 10.3389/fpls.2023.1184058

**Published:** 2023-06-21

**Authors:** Zhou Zhang, Sunil S. Gangurde, Songbin Chen, Rushil Ramesh Mandlik, Haiyan Liu, Rupesh Deshmukh, Jialing Xu, Zhongkang Wu, Yanbin Hong, Yin Li

**Affiliations:** ^1^ Guangdong Key Laboratory of Plant Resources, School of Life Sciences, Sun Yat-sen University, Guangzhou, China; ^2^ Department of Plant Pathology, University of Georgia, Tifton, GA, United States; ^3^ Department of Agriculture Biotechnology, National Agri-food Biotechnology Institute (NABI), Mohali, India; ^4^ Crops Research Institute, Guangdong Academy of Agricultural Sciences, Guangzhou, China

**Keywords:** *Arachis hypogaea*, 14-3-3, GRF, transgenic *Arabidopsis thaliana*, root, auxin signaling

## Abstract

The 14-3-3 protein is a kind of evolutionary ubiquitous protein family highly conserved in eukaryotes. Initially, 14-3-3 proteins were reported in mammalian nervous tissues, but in the last decade, their role in various metabolic pathways in plants established the importance of 14-3-3 proteins. In the present study, a total of 22 *14-3-3* genes, also called general regulatory factors (*GRF*), were identified in the peanut (*Arachis hypogaea*) genome, out of which 12 belonged to the ε group, whereas 10 of them belonged to the non- ε-group. Tissue-specific expression of identified *14-3-3* genes were studied using transcriptome analysis. The peanut *AhGRFi* gene was cloned and transformed into *Arabidopsis thaliana*. The investigation of subcellular localization indicated that *AhGRFi* is localized in the cytoplasm. Overexpression of the *AhGRFi* gene in transgenic *Arabidopsis* showed that under exogenous 1-naphthaleneacetic acid (NAA) treatment, root growth inhibition in transgenic plants was enhanced. Further analysis indicated that the expression of auxin-responsive genes *IAA3*, *IAA7*, *IAA17*, and *SAUR-AC1* was upregulated and *GH3.2* and *GH3.3* were downregulated in transgenic plants, but the expression of *GH3.2*, *GH3.3*, and *SAUR-AC1* showed opposite trends of change under NAA treatment. These results suggest that *AhGRFi* may be involved in auxin signaling during seedling root development. An in-depth study of the molecular mechanism of this process remains to be further explored.

## 1 Introduction

In multicellular organisms, the exchange of information via signaling within the cell and between cells can stimulate the recognition of biotic and abiotic factors so that the plant system can respond to stresses and regulate growth and development. The growth and genetic potential of a plant are affected by a variety of environmental factors such as light, temperature, humidity, moisture, lodging, wind velocity, and the internal plant atmosphere, which includes plant hormones, signaling molecules, chlorophyll content, and stomatal movement (Fulgosi et al., 2002). There are small molecular proteins called 14-3-3 proteins in plant cells that play an important role in cell signal transduction in response to biotic and abiotic stresses and regulation of plant growth and development (Grienenberger and Fletcher, 2015; Hajibarat et al., 2022; Huang et al., 2022). In addition, it also participates in material transport and metabolism during various metabolic processes and plant hormone signaling. These signaling proteins interact with plant defense protein factors, and kinase activation also plays an important role in the inhibition of cell division and differentiation and oil transport proteins. The 14-3-3 proteins, which are phosphorylation-dependent or phosphorylation-independent binding protein, also stimulate gibberellin transcription protein (GASA), etc. (Herzog et al., 1995; Hughes and Dunn, 1996; Boutrot et al., 2008; Marshall et al., 2011). 14-3-3 proteins act as hubs for a cellular web, transducing and integrating diverse hormone signals (Camoni et al., 2018). Moreover, plant 14-3-3 proteins are also considered to be indirectly involved in auxin signaling through the protein kinase pathway (Takahashi et al., 2012).

The 14-3-3 proteins were initially cataloged by Moore and Perez (1967) during an extensive study of bovine brain proteins. The name 14-3-3 proteins originated from phase chromatography using diethylaminoethyl cellulose (DEAE) fraction elution in the position of fragments and starch migration rate in gel electrophoresis. Further, studies on 14-3-3 proteins demonstrated that these proteins are highly conserved in eukaryotes and form heterologous or homologous dimeric molecules with monomeric protein molecules. The 14-3-3 proteins were extensively studied in Arabidopsis and are also known as *GF14*s or general regulatory factors (*GRFs*) (Rooney and Ferl, 1995; Wu et al., 1997). A varying number of 14-3-3 genes are identified in the genomes of different plant species, for example, 10 in Arabidopsis and 18 in soybean, which is a polyploid plant species (Wu et al., 1997; Li and Dhaubhadel, 2011).

The 14-3-3 interacts with target proteins through a specific phosphorylation site, resulting in protein–protein interactions and regulating signal transduction (Denison et al., 2011; Paul et al., 2012). In plants, more than 300 target proteins were identified to interact with the 14-3-3 protein (Oecking and Jaspert, 2009). These target proteins are involved in a variety of important physiological processes during abiotic and biotic stress tolerance, activity and stability confirmation of proteins, protein localization, and transport and regulation of cell division (Yang et al., 2014). There are several reports on the expression and functional diversity of H^+^-ATPase membrane proteins. The 14-3-3 protein is involved in the regulation of the activity of H^+^-ATPase (Duby et al., 2008). Nitrate reductase is a key nitrate metabolic enzyme. The activity of nitrate reductase is dependent on the process of phosphorylation. In the dark, 14-3-3 protein inhibits the activity of nitrate reductase and reduces the production and metabolism of toxic nitrate (Bachmann et al., 1996). Sucrose phosphate synthase is a key enzyme in carbon metabolism in plants; the 14-3-3 can inhibit the activity of sucrose phosphate synthase. On the other hand, with the inhibition of 14-3-3 activity, the corresponding starch and sucrose content increases (Moorhead et al., 1999; Toroser et al., 2000). In barley, 14-3-3 protein interacts with transcription factors such as *AREB*, *ABF/ABI5*, which are involved in abscisic acid (ABA) metabolism. The *VP1* gene mutations in *ABI5* cannot be combined without 14-3-3 proteins; studies reported that binding *VP1* and *ABI5* needs 14-3-3 as a bridge to form a complex binding to the ABA-responsive element (Himmelbach et al., 2003). 14-3-3 negatively regulates *BRZ1* and *BRZ2*/*BES1* transcription in *Arabidopsis thaliana* and rice (Bai et al., 2007; Gampala et al., 2007). The 14-3-3 protein is also involved in the signaling of brassinosteroids (Wang et al., 2011) and gibberellins (Ishida et al., 2004), using a photographic approach (Taoka et al., 2011). Plant 14-3-3 proteins and target protein binding during the stress response stimulate signaling mechanisms in transgenic plants, which include the non-biological responses to the ROS reaction, salt stress tolerance, osmotic stress tolerance, and high and low-temperature stress responses (Kidou et al., 1993; Chen et al., 1994; Roberts et al., 2002; Elmayan et al., 2007). In addition, the biological response of plant resistance genes (R-genes) in response to pathogen attack (Yang et al., 2009). Peanuts are an important oilseed and legume crop. Most of the research efforts in peanut are carried out to improve the resistance against devastating biotic stresses such as stem rot (Dodia et al., 2019), bacterial wilt (Luo et al., 2019) and late leaf spot (Gangurde et al., 2021), bacterial wilt (Luo et al., 2019). Around 90% of peanuts are cultivated in semi-arid zones of the world where drought is an important yield-limiting factor (Gangurde et al., 2019; Pandey et al., 2021). As discussed above, several reports have revealed that the *14-3-3* gene involves the regulation of growth and development and the stimulation of responses to biotic and abiotic stresses in plants. Therefore, it is necessary to perform structural and functional analysis of the peanut *14-3-3* gene so that it can be used as a molecular tool to improve peanut varieties resistant to biotic and abiotic stresses. In this context, in the present study, 22 *14-3-3* genes, named *AhGRF*s, were identified through a genome-wide identification approach. Among the identified genes, *AhGRFi* was cloned and transformed into *A. thaliana* to analyze its function. The sequence similarity of the peanut *14-3-3* protein was investigated, and the expression pattern of the *14-3-3* genes in different plant tissues of peanut during different developmental stages was studied. The expression of the *14-3-3* gene was validated using quantitative real-time quantitative PCR.

## 2 Materials and methods

### 2.1 Genome-wide identification of AhGRF proteins in peanut

The genome information for peanut (*Arachis hypogaea* v1.0) was obtained from the phytozome database (Goodstein et al., 2012) at https://phytozome-next.jgi.doe.gov/. A local protein database was created in BioEdit (Hall et al., 2011) using protein sequences obtained from the phytozome database (Ahypogaea_530_v1.0). Previously identified 14-3-3 proteins from rice (Yashvardhini et al., 2018), soybean (Li and Dhaubhadel, 2011), and Arabidopsis (Wu et al., 1997) were used as a query for BLASTp against the local protein database for peanut. To identify significant candidates, the minimum cut-off value was kept to E-value 10^−5^ and a bit score >100. The top-hit candidates with the highest bit score were selected, and redundant ones were filtered. The non-redundant candidates were used for further analysis. A genetic map was generated by the online tool MG2C (http://mg2c.iask.in/mg2c_v2.0). The identified AhGRF proteins were further confirmed through HMMER (https://www.ebi.ac.uk/Tools/hmmer/) and the Pfam database (https://pfam.xfam.org/).

The molecular weight and isoelectric focusing point for the identified proteins were estimated by using Expasy tools (https://web.expasy.org/compute_pi/). The TBtools (https://bio.tools/tbtools) were used to predict the gene structure. The subcellular localization of the identified protein was predicted using CELLO (http://cello.life.nctu.edu.tw/) and Wolfpsort (https://wolfpsort.hgc.jp/) servers. The transmembrane domains were predicted using TMHMM (http://www.cbs.dtu.dk/services/TMHMM/) and SOSUI Server (http://harrier.nagahama-i-bio.ac.jp/sosui/). The motifs were identified by the MEME tool (http://meme-suite.org/tools/meme).

### 2.2 Multiple sequence alignment and phylogenetic analysis

Multiple sequence alignments for peanut AhGRF proteins were performed in the CLUSTAL W function in MEGA X. The phylogenetic tree was generated based on 14-3-3 protein sequence alignment via the MEGA X program using the maximum likelihood method. The robustness of the tree was confirmed by using a 1,000-bootstrap value.

### 2.3 Expression analysis of *AhGRF* genes

The RNA-seq data for expression analysis of 22 *AhGRF* genes were downloaded from the NCBI Sequence Read Archive (Bio Project IDs: PRJNA243319, PRJNA773958, PRJNA687108, PRJNA638812, PRJNA555172, PRJNA525247, PRJNA511663, PRJNA517600, PRJNA503795, PRJNA498570, and PRJNA291488). The CLC workbench (https://www.qiagenbioinformatics.com/products/clc-genomics-workbench/) was used to process the raw reads. The reads were mapped onto the peanut reference genome sequence. The reads per kilobase of the transcript per million (RPKM) mapped read values were calculated, and a heatmap for the same was generated using the R package ‘pheatmap’ (Kolde and Kolde, 2018). Hierarchical clustering was utilized to cluster genes.

### 2.4 Plant and growth condition

The cultivated peanut (*A. hypogaea* L.) variety of Shanyou 523 seeds was provided by the Guangdong Academy of Agricultural Sciences, Guangzhou, China. *A. thaliana* (Columbia-0 Ecotype) was used for peanut *AhGRFi* gene transformation and functional characterization. The seeds of transgenic *A. thaliana* were sterilized with 70% (v/v) ethyl alcohol for 2 min and then transferred to a 10% (v/v) sodium hypochlorite solution for 10 min. Further, the seeds were rinsed with sterile water five times on a clean bench. *A. thaliana* seeds were placed on one-half MS solid culture medium (pH 5.8, 1 mM KOH) after surface sterilization (Murashige and Skoog, 1962) and incubated at 4°C temperature for 3 days. They were further grown at 22°C with a 16 h/8 h photoperiod and 6,000 lx light intensity (lux). After two weeks, seedlings were transplanted to the soil for growth and maturation under the same growth conditions (peat soil:vermiculite ratio 3:1).

### 2.5 Gene cloning and sequence analysis

The full-length cDNA sequence of the *AhGRFi* gene, which is identical to that of LOC112798274 14-3-3-like protein A in *A. hypogaea* with Gene ID: 112798274 in the gene database of the National Center for Biotechnology Information (NCBI) (https://www.ncbi.nlm.nih.gov/gene/112798274), was amplified using gene-specific primers (**Table S1**) from the peanut cotyledons cDNA library (Li et al., 2005). The *AhGRFi* amplicon was extracted and purified from agarose gel using a quick gel extraction kit (QIAGEN) as per the manufacturer’s instructions. The purified *AhGRFi* gene cDNA sequence was cloned into the pMD18-T vector for sequencing.

### 2.6 Peanut *AhGRFi* protein interaction experiment *in vitro*

The yeast two-hybrid assay was performed according to the manufacturer’s instructions for the Matchmaker GAL4-based two-hybrid system 3 (Clontech) (Zhou et al., 2013). The full-length *AhGRFi* cDNA was subcloned into the pGADT7 (AD) and pGBKT7 (BD) vectors. All primer sequences used for construction are listed in **Table S1**. Constructs were transformed into yeast strain AH109 by the lithium acetate method, and yeast cells were grown on a minimal medium/-Leu-Trp at 30°C for 3 days. Transformed colonies were plated onto a minimal medium/-Leu/-Trp/-His/-Ade containing 20 µg/ml 5-bromo-4-chloro-3-indolyl-a-D-galactopyranoside to test for possible interactions.

### 2.7 Analysis of the expression pattern of *AhGRFi* gene in peanut

Total RNA isolation was carried out from the roots, stems, and leaves of the 12-day-old seedling and the 20, 30-, 40-, 50-, and 70-day-old embryos after pegging into soil (DAP) using the TRIZOl reagent (Invitrogen). The cDNA was synthesized from isolated RNA using the PrimeScript 1st Strand cDNA Synthesis Kit (TaKaRa).

Expression patterns of the *AhGRFi* gene in peanut tissues were investigated using qRT-PCR, according to Li et al. (2009). The qRT-PCR was performed on the LightCycler480 (RoChe) using the SYBR Green real-time PCR Master Mix (Toyobo) with gene-specific primers and 18S rRNA (Guo et al., 2012) as the internal control gene. Primer sequences used for this analysis are listed in **Table S1**.

### 2.8 Subcellular localization of *AhGRFi* protein

The coding region without a stop codon for the *AhGRFi* gene from peanut was cloned into the intermediate vector pA7-YFP, and the *AhGRFi : YFP* cassette was further subcloned into the pBI121 vector, replacing the *GUS* gene. Primer sequences used for the construction are listed in **Table S1**. The recombinant vector was transformed into *Agrobacterium tumefaciens* strain EHA105. Tobacco (*Nicotiana tabacum* var. Xanthi) leaf epidermal cells were infiltrated with *Agrobacterium* carrying a recombinant vector with *35Spro : AhGRFi-YFP*. Simultaneously, in the control experiment, the tobacco epidermal cells were infiltrated with *Agrobacterium* (*35Spro : YFP).* The infiltrated plants were maintained at under 22°C with 16 h of photoperiod, and the light intensity was 6,000 lx. The YFP signal was subsequently detected by the LSM 710 duo laser scanning confocal microscope (ZEISS, Germany) with a filter set of 514 nm for excitation and 527 nm for emission after 24 h of agroinfiltration.

### 2.9 Construction of *AhGRFi* overexpression vector and *A. thaliana* transformation

For the heterologous expression, the coding sequence of *AhGRFi* was cloned into the pBI121 vector under the control of the CaMV 35S promoter. The recombinant plasmid was transformed into the *Agrobacterium tumefaciens* EHA105 strain. The recombinant plasmid was transformed into *A. thaliana* by the floral dip method (Clough and Bent, 1998). Transgenic seeds were selected on a one-half MS medium containing 50 mg/L kanamycin. According to the segregation ratio of the kanamycin selection marker, homozygous transgenic lines for *Arabidopsis* were identified and further confirmed by leaf PCR (Kasajima et al., 2004) using gene-specific primers of *AhGRFi*.

### 2.10 Phenotype analysis of transgenic *A. thaliana*

Seeds of transgenic *Arabidopsis* carrying the *AhGRFi* gene and wild-type *Arabidopsis* were surface sterilized and placed on one-half MS medium containing 1 uM Naphthalene acetic acid (NAA) and incubated at 4°C for 48 h under normal environmental conditions at 22°C at 16 h light/8 h dark, vertical 6,000 lx for vertical training for 2 weeks. Later, the root length was recorded for both control and transgenic *Arabidopsis* to study the effect of the *AhGRFi* gene on root growth. Three biological repeats were carried out for each treatment; 15 plants were used each time. Statistical analyses were performed using the Student’s *t*-test. All data were expressed as means ± standard error (SE). p <0.05 and p <0.01 are considered statistically significant.

### 2.11 qRT-PCR analysis of transgenic *Arabidopsis*

Total RNA was isolated from Arabidopsis seedlings cultured on one-half MS medium for 2 weeks using the RNAprep Pure Plant Kit (TIANGEN). The cDNA was synthesized from isolated RNA using ReverTra Ace qPCR RT Master Mix with gDNA Remover (Toyobo). Real-time PCR analysis of overexpression in transgenic lines was performed with specific primers for *AhGRFi* and with *Actin2* and *UBQ10* as internal control genes. Auxin-responsive gene expression in transgenic *Arabidopsis* was analyzed with gene-specific primers and the *Actin2* gene as an internal control (Yang et al., 2014). Primer sequences are shown in **Table S1**.

## 3 Results

### 3.1 Genome-wide identification of *AhGRF* proteins in peanut

Based on sequence homology of previously characterized 14-3-3 protein-encoding genes in rice, soybean, and *Arabidopsis* a total of 22 *14-3-3* genes were identified in the peanut genome (**Table 1**). A genetic map was generated by MG2C, showing the overall distribution of *AhGRF* gene members and their corresponding positions on chromosomes (**Figure S1**). HMMER and Pfam confirmed the presence of 14-3-3 domains in the identified gene-encoded proteins. The identified genes encode proteins ranging from 196 to 300 amino acids. Their molecular weight ranged from 22,103 to 34,357 Da, while their isoelectric point ranged from 4.4 to 4.8. The TMHMM and SOSUI servers confirmed the absence of transmembrane domains in peanut 14-3-3 proteins. The genes *AhGRFi* and *AhGRFj*, *AhGRFo*, and *AhGRFp* encoded similar proteins with identical amino acid sequences.

**Table 1:** The *14-3-3* genes identified in peanut.

Name	Gene id	Chromosome	Location	Genomic sequence (bp)	Transcription(bp)	CDS(bp)
***AhGRFa***	arahy.Tifrunner.gnm1.ann1.ZP68QF	17	51704361–51707007 (−)	2,647	1,535	786
***AhGRFb***	arahy.Tifrunner.gnm1.ann1.3WX4S3	7	59732514–59735109 (+)	2,596	1,544	786
***AhGRFc***	arahy.Tifrunner.gnm1.ann1.37SQSE	8	13351021–13354211 (+)	3,191	2,454	918
***AhGRFd***	arahy.Tifrunner.gnm1.ann1.PE54XI	17	130076384–130079623 (+)	3,240	2,366	780
***AhGRFe***	arahy.Tifrunner.gnm1.ann1.5WU7C9	14	135871730–135874228 (+)	2,499	825	825
***AhGRFf***	arahy.Tifrunner.gnm1.ann1.L8VLW8	4	121555458–121557140 (+)	1,683	825	825
***AhGRFg***	arahy.Tifrunner.gnm1.ann1.58PDLX	17	130872178–130876189 (−)	4,012	1,401	864
***AhGRFh***	arahy.Tifrunner.gnm1.ann1.JWB9EG	8	14151438–14155456 (−)	4,019	1,272	864
***AhGRFi***	arahy.Tifrunner.gnm1.ann1.840CAR	14	142826581–142836826 (+)	10,246	8,371	783
***AhGRFj***	arahy.Tifrunner.gnm1.ann1.ZQ326X	4	128391051–128401296 (+)	10,246	8,371	783
***AhGRFk***	arahy.Tifrunner.gnm1.ann1.B783NX	13	138434784–138437897 (+)	3,114	1,471	840
***AhGRFl***	arahy.Tifrunner.gnm1.ann1.YHZ4YG	3	135571591–135574921 (+)	3,331	1,415	588
***AhGRFm***	arahy.Tifrunner.gnm1.ann1.FHP35I	15	77667261–77670435 (+)	3,175	777	777
***AhGRFn***	arahy.Tifrunner.gnm1.ann1.P3KMTI	5	63712889–63730327 (−)	17,439	1,005	900
***AhGRFo***	arahy.Tifrunner.gnm1.ann1.CC9DVG	8	34958465–34961268 (−)	2,804	1,759	786
***AhGRFp***	arahy.Tifrunner.gnm1.ann1.3M7PWZ	12	7927632–7930442 (+)	2,811	1,766	786
***AhGRFr***	arahy.Tifrunner.gnm1.ann1.HQ7EPF	7	78994675–78998944 (+)	4,270	1,314	792
***AhGRFs***	arahy.Tifrunner.gnm1.ann1.I3F13H	18	29271174–29275502 (−)	4,329	1,326	792
***AhGRFt***	arahy.Tifrunner.gnm1.ann1.2XH6EA	13	145842650–145845795 (+)	3,146	1,653	849
***AhGRFq***	arahy.Tifrunner.gnm1.ann1.0DN2NX	3	142965807–142968931 (+)	3,125	1,560	780
***AhGRFu***	arahy.Tifrunner.gnm1.ann1.9B275P	20	3717468–3721401 (−)	3,934	1,090	855
***AhGRFv***	arahy.Tifrunner.gnm1.ann1.WH2NDV	10	1623462–1626412 (−)	2,951	1,800	771

### 3.2 Phylogenetic and evolutionary analysis of *AhGRF* proteins in peanut

**Figure 1:**
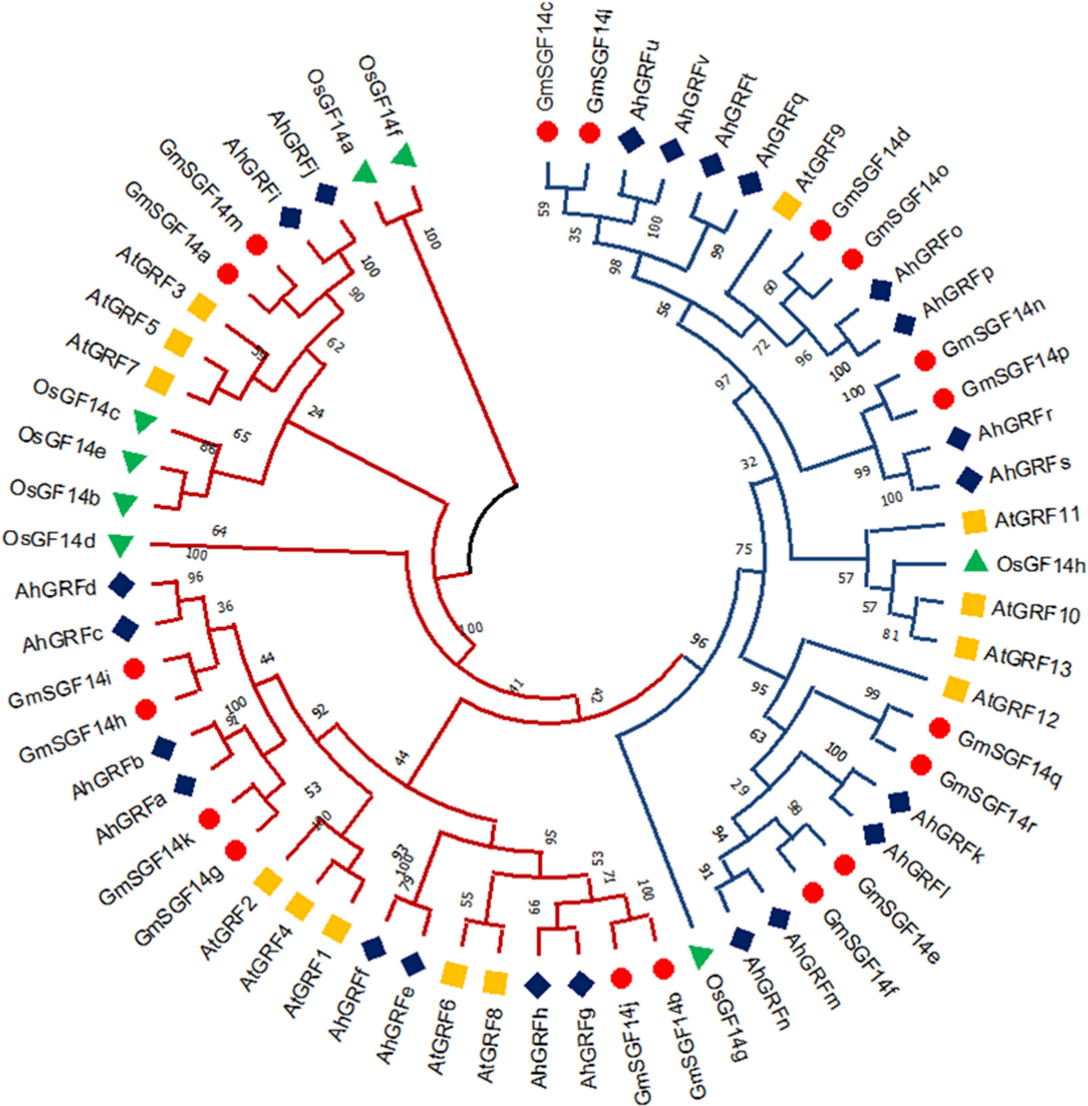
Phylogenetic tree showing the evolutionary relationship among AhGRF proteins identified in peanut, soybean, *Arabidopsis*, and rice.

**Figure 2:**
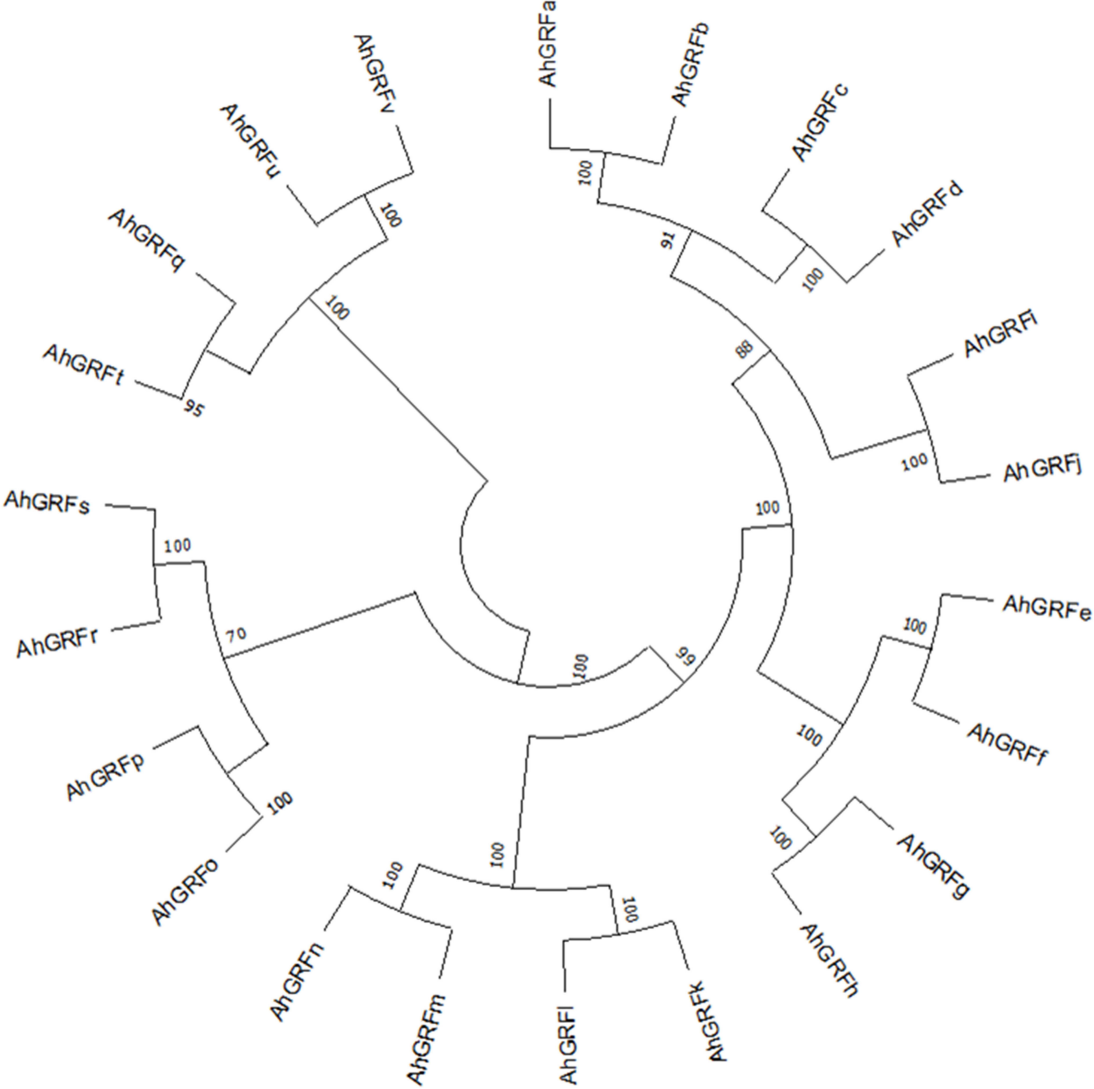
Neighbor-joining phylogenetic tree of AhGRF proteins identified in peanut.

The phylogenetic tree generated using the maximum likelihood method in the MEGA X program gives insight into the evolution pattern of peanut *AhGRF* genes. The phylogenetic tree was constructed using the *AhGRF* protein sequences from peanut, rice, soybean, and Arabidopsis (**Figure 1**). As per previous studies, the 14-3-3 proteins were divided into ε and non-ε groups. The AhGRFu, AhGRFv, AhGRFq, AhGRFo, AhGRFp, AhGRFr, AhGRFs, AhGRFk, AhGRFl, AhGRFm, AhGRFn, and AhGRFt constituted the ε group, whereas AhGRFd, AhGRFi, AhGRFj, AhGRFc, AhGRFb, AhGRFa, AhGRFf, AhGRFe, AhGRFh, and AhGRFg belonged to the non-ε group. Both the ε and non-ε groups were divided into four branches, which were subsequently divided into further subbranches. The gene pairs *AhGRFi* and *AhGRFj* and *AhGRFo* and *AhGRFp* were closely related and encoded for similar proteins with identical amino acid sequences (**Figure 2**, **Table 2**). The similarity between the respective gene sequence pairs was found to be 100%.

**Table 2:** Physical properties of 14-3-3 proteins identified in peanut.

Protein	Protein id	Peptide length (aa)	Molecular weight (da)	Average isoelectric point (PI)	Protein isoelectric point (IPC)
**AhGRFa**	arahy.Tifrunner.gnm1.ann1.ZP68QF.1	262	29,348.81994	4.536	4.649
**AhGRFb**	arahy.Tifrunner.gnm1.ann1.3WX4S3.1	262	29,308.79564	4.529	4.649
**AhGRFc**	arahy.Tifrunner.gnm1.ann1.37SQSE.1	306	34,538.02184	4.498	4.611
**AhGRFd**	arahy.Tifrunner.gnm1.ann1.PE54XI.1	260	29,199.69744	4.482	4.592
**AhGRFe**	arahy.Tifrunner.gnm1.ann1.5WU7C9.1	275	30,711.56154	5.147	5.238
**AhGRFf**	arahy.Tifrunner.gnm1.ann1.L8VLW8.1	275	30,559.34394	4.785	4.901
**AhGRFg**	arahy.Tifrunner.gnm1.ann1.58PDLX.1	288	32,104.29024	4.779	4.896
**AhGRFh**	arahy.Tifrunner.gnm1.ann1.JWB9EG.1	288	32,078.20904	4.779	4.896
**AhGRFi**	arahy.Tifrunner.gnm1.ann1.840CAR.2	261	29,405.95884	4.51	4.631
**AhGRFj**	arahy.Tifrunner.gnm1.ann1.ZQ326X.2	261	29,405.95884	4.51	4.631
**AhGRFk**	arahy.Tifrunner.gnm1.ann1.B783NX.1	280	31,747.84104	4.825	4.961
**AhGRFl**	arahy.Tifrunner.gnm1.ann1.YHZ4YG.1	196	22,103.86474	4.595	4.722
**AhGRFm**	arahy.Tifrunner.gnm1.ann1.FHP35I.1	259	29,217.16514	4.709	4.82
**AhGRFn**	arahy.Tifrunner.gnm1.ann1.P3KMTI.1	300	34,357.95694	4.715	4.838
**AhGRFo**	arahy.Tifrunner.gnm1.ann1.CC9DVG.1	262	29,481.00644	4.611	4.731
**AhGRFp**	arahy.Tifrunner.gnm1.ann1.3M7PWZ.1	262	29,481.00644	4.611	4.731
**AhGRFr**	arahy.Tifrunner.gnm1.ann1.HQ7EPF.1	264	30,069.76924	4.594	4.703
**AhGRFs**	arahy.Tifrunner.gnm1.ann1.I3F13H.1	264	30,027.83354	4.608	4.717
**AhGRFt**	arahy.Tifrunner.gnm1.ann1.2XH6EA.1	283	31,933.06064	4.75	4.871
**AhGRFq**	arahy.Tifrunner.gnm1.ann1.0DN2NX.1	260	29,280.82714	4.587	4.709
**AhGRFu**	arahy.Tifrunner.gnm1.ann1.9B275P.1	285	31,924.80974	4.564	4.682
**AhGRFv**	arahy.Tifrunner.gnm1.ann1.WH2NDV.1	257	28,924.54074	4.634	4.754

**Figure 3:**
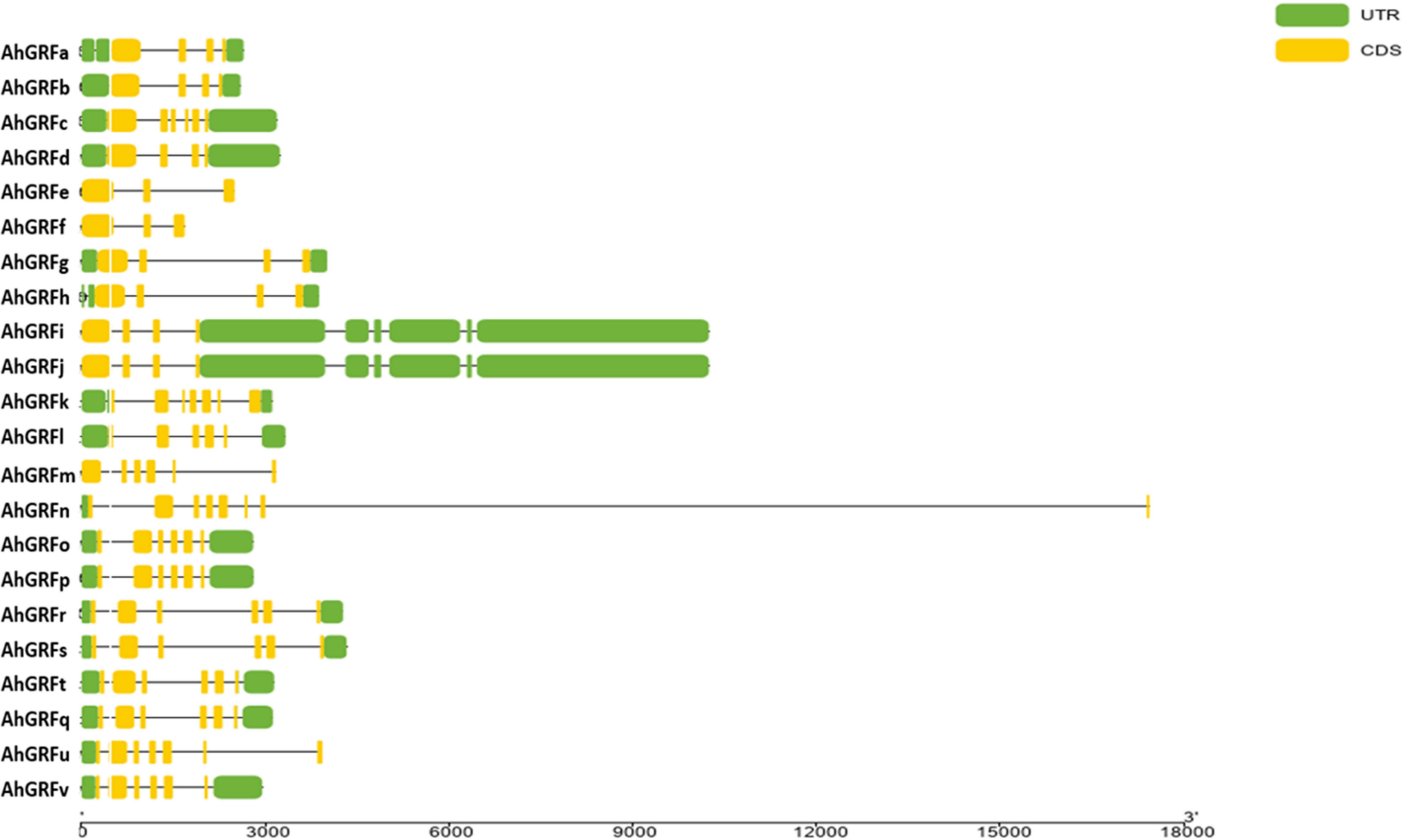
Intron–exon structure for AhGRF proteins identified in peanut.

The number of introns varied from two to 10 in *AhGRF* genes identified in peanuts. Some of the gene pairs displayed a similar number of introns and exons, which were also similar in length. *AhGRFi* and *AhGRFj*, *AhGRFo*, and *AhGRFp* genes encoded for identical proteins also have similar gene structures with the same number of introns and exons (**Figure 3**).

### 3.3 Expression analysis of *AhGRF* genes in different tissues and stress conditions

**Figure 4:**
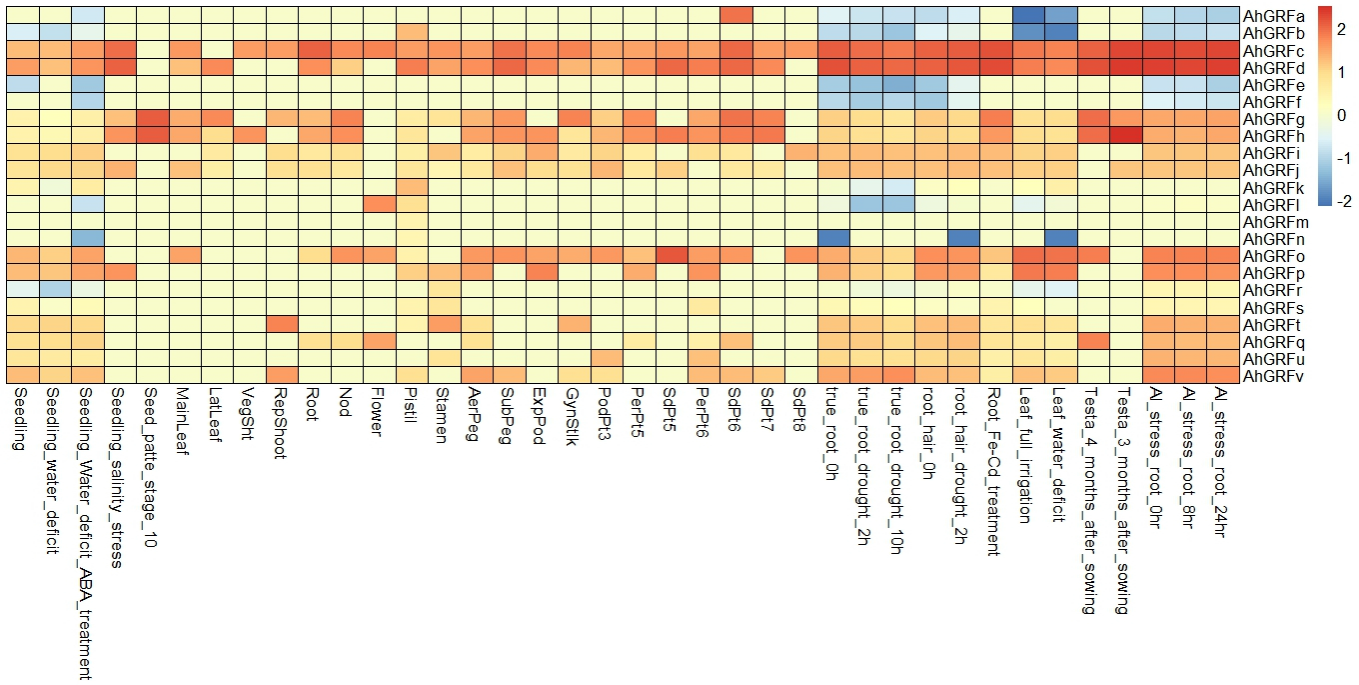
Analysis of the expression pattern of the *AhGRF* genes.

The expression of the identified *AhGRF* genes was determined using transcriptomic data from various developmental stages and stress conditions. The genes *AhGRFe* and *AhGRFf* showed expression in almost all tissues except reproductive tissues. The expressions of *AhGRFk*, *AhGRFb*, *AhGRFm*, and *AhGRFl* were found to be specific to the pistil and flower, respectively. The *AhGRFh* was found to be mainly expressed in seed and pod tissues, while the *AhGRFr* was primarily expressed in peg tissues. In comparison, *AhGRFp* expression was restricted to the testa, roots, and some seed tissues, whereas *AhGRFi* expression was restricted to leaf, root, and some pod tissues. The *AhGRFj* is primarily expressed in the testa, roots, pod, and lateral tissues. The *AhGRFs, AhGRFu, and AhGRFt* were primarily expressed in the root and some of the pod tissues only. Aluminum stress did not show any significant change in root gene expression. Iron–cadmium treatment showed expression of *AhGRFg*, *AhGRFc*, *AhGRFd*, and *AhGRFs* in the roots. A water deficit increased the expression of *AhGRFi* and *AhGRFk* in leaf tissue. While drought stress at the seedling stage increased the expression of *AhGRFe*, *AhGRFf*, and *AhGRFt* genes while reducing the expression of *AhGRFr*, *AhGRFc*, and *AhGRFd* (**Figure 4**).

### 3.4 Cloning of *AhGRFi* gene

In a previous analysis of a peanut cotyledon cDNA library (Li et al., 2005), a 14-3-3-like protein gene sequence was identified. Our further cloning and analysis found that it was an *AhGRFi* gene identical to that of LOC112798274 14-3-3-like protein A with Gene ID: 112798274 in the NCBI gene database. In this study, a 944-bp sequence containing the 783-bp full CDS of the *AhGRFi* gene was amplified from the corresponding peanut cDNA library using gene-specific primers by PCR (**Figure S2**). The resulting *AhGRFi* sequence was cloned by the T-A cloning methodology into the pMD18-T vector and sequenced for confirmation of the presence of the target gene.

### 3.5 Expression analysis of *AhGRFi* gene by qRT-PCR

**Figure 5:**
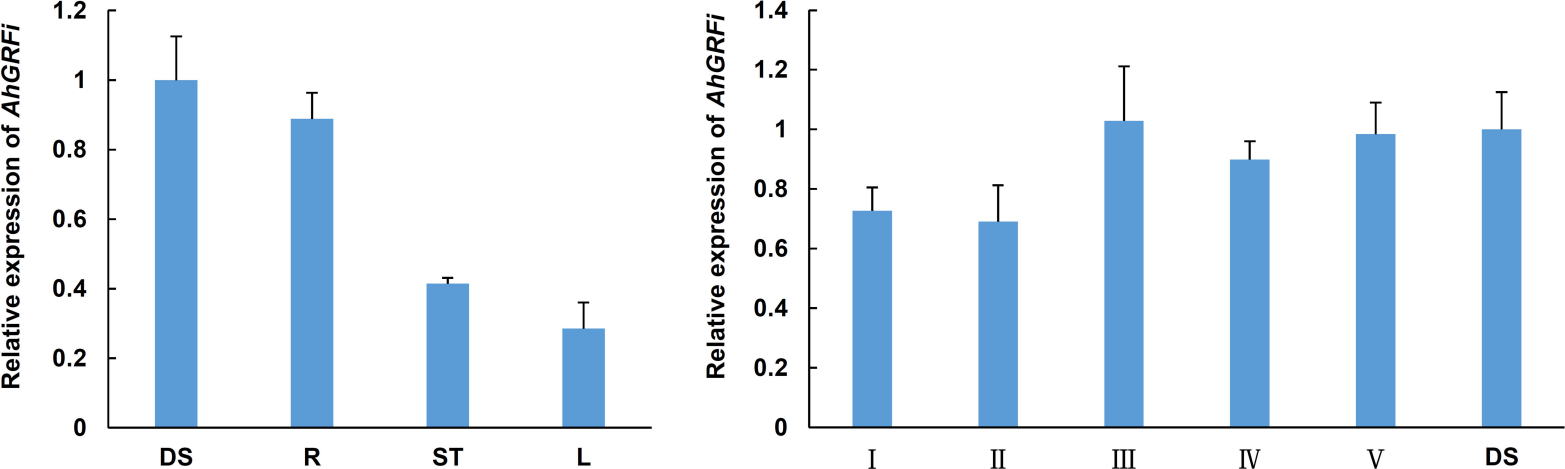
Analysis of the expression pattern of the *AhGRFi* gene. Error bars represent the means ± SE of three replications. Different organizations of peanut RNA: DS, dry seeds; R, stem; L, leaf; Different development periods of peanut RNA: I, 20 DAP; II, 30 DAP; III, 40 DAP; IV, 50 DAP; V, 70 DAP; *Ah18S RNA* reference gene. Error bars represent the means ± SE of three replications.

To understand the role of the *AhGRFi* gene in peanut growth and development, RNA was extracted from different peanut developmental stages and tissues, and the expression pattern of the *AhGRFi* gene was studied using real-time fluorescent quantitative PCR (**Figure 5**). The *AhGRFi* showed the highest expression level in peanut seeds and seedling roots. In different developmental stages of peanut seeds, the expression of *AhGRFi* was relatively stable, and the expression level at 10 d and 20 d after the pegging stage was slightly lower as compared to the later stages.

### 3.6 Interaction of *AhGRFi in vitro*

**Figure 6:**
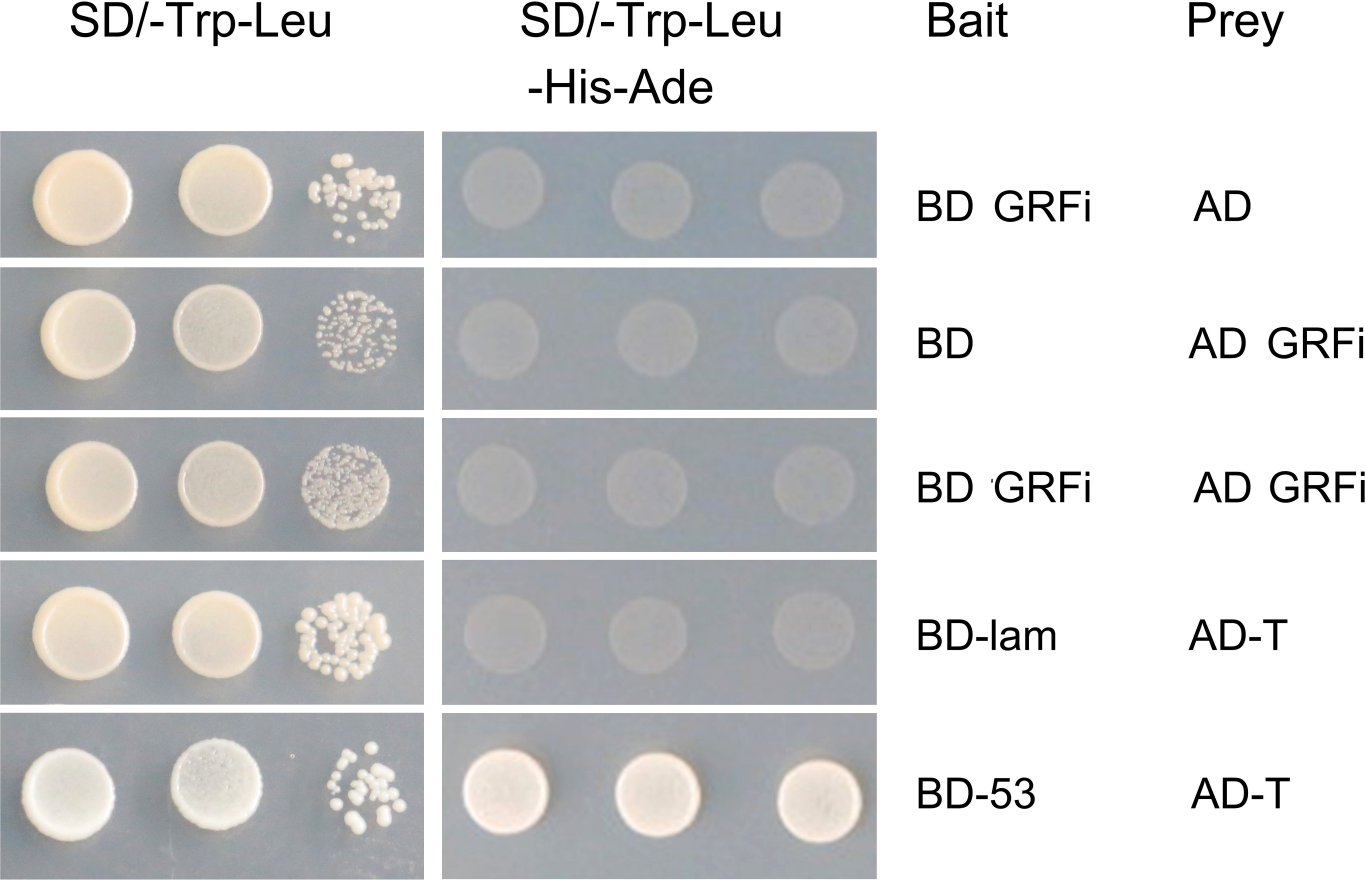
AhGRFi protein interaction analyses *in vitro*. Interaction was determined by growth assays on medium lacking adenine. Dilutions (1.10^−1^, and 10^−2^ for control and without dilution for lacking adenine) of saturated cultures were spotted onto the plates.

In eukaryotic cells, 14-3-3 proteins are usually bound to two target proteins to function as homologous or heterologous dimers. To understand the interaction mechanism of the peanut AhGRFi protein, the *AhGRFi* gene was cloned into pGADT7 and pGBKT7 vectors and transformed into yeast-competent cells (AH109). The yeast could grow in the SD/-Trp-Leu medium, but the colony in the SD/-Trp-Leu medium did not grow in the SD/-Trp-Leu-His-Ade medium (**Figure 6**). *In vitro* experimental results showed that the peanut protein AhGRFi did not interact with itself, suggesting that the AhGRFi protein does not function as a homodimer.

### 3.7 *AhGRFi* protein is localized in the cytoplasm

**Figure 7:**
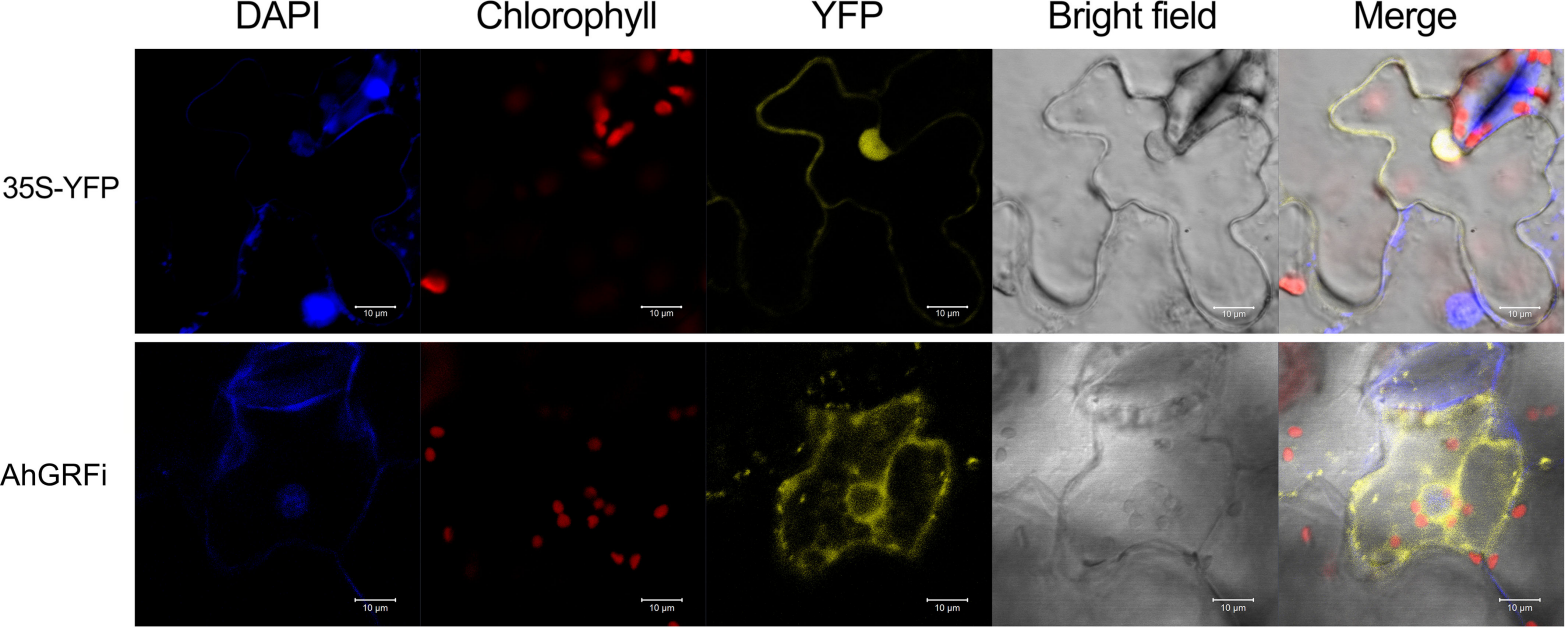
Subcellular localization of *AhGRFi* in tobacco epidermal cells.

To investigate the subcellular localization of AhGRFi, YFP (yellow fluorescent protein)-tagged AhGRFi, driven by the CaMV 35S promoter, was agroinfiltrated into tobacco epidermal cells to express the fusion protein. The AhGRFi : YFP fusion proteins were examined with a Zeiss confocal laser scanning microscope (at 514 nm for the excitation spectra and at 527 nm for the emission spectra). We observed that the fluorescence signal was mainly distributed in the cytoplasm, as shown in **Figure 7**. The coincidence of yellow fluorescent protein signals in the nucleus was confirmed by DAPI staining. Therefore, we concluded that the peanut AhGRFi protein is mainly distributed in the cytoplasm and not in the nucleus.

### 3.8 Overexpression of *AhGRFi* in *Arabidopsis* conferred sensitive to NAA

**Figure 8:**
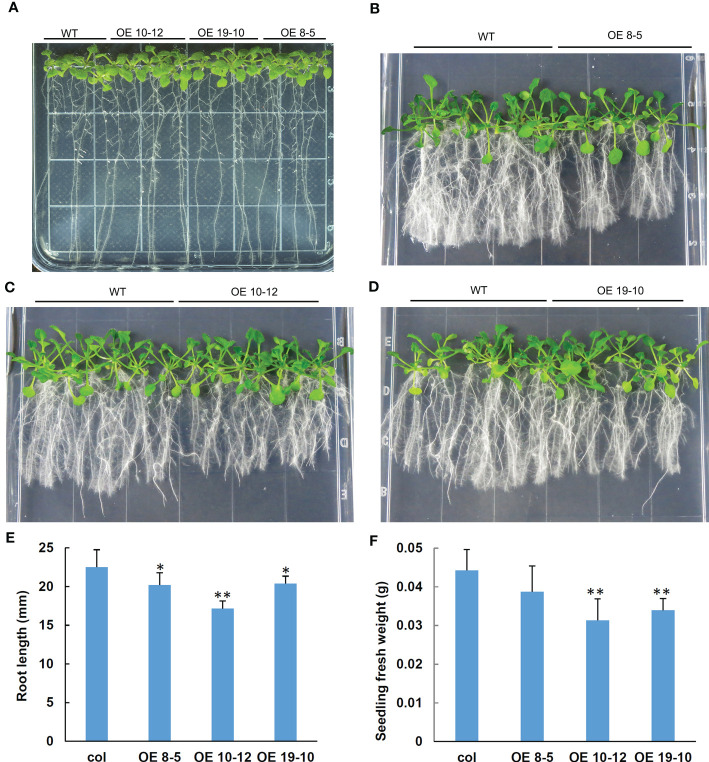
Effect of NAA on root lengths of transgenic *Arabidopsis* overexpressing *AhGRFi*. **(A)** Phenotypes of transgenic *Arabidopsis* in one-half MS medium under NAA treatment **(A)** control group; **(B–D)** treatment group). Measurement of the *Arabidopsis* root length **(E)** and fresh weight of the *Arabidopsis* seedlings **(F)** Error bars represent the means (n = 15) ± SE of three replications. * and ** above the error bars indicate statistical significance at P <0.05 and P <0.01, respectively.

To study the biological function of peanut *AhGRFi*, it was heterologously expressed in *A. thaliana* via *Agrobacterium-*mediated transformation. Transgenic lines were confirmed by PCR (**Figure S3**). qRT-PCR was performed to detect the overexpression levels in transgenic lines, and three high-expression lines were used for further study (**Figure S4**). Under normal culture conditions, when transgenic *A. thaliana* with overexpression of the *AhGRFi* gene was compared with a wild-type control, there was no difference in *Arabidopsis* growth and seed development in both overexpression and wild-type *Arabidopsis* lines (**Figure 8A**). In the presence of exogenous NAA treatment, two-week-old overexpression lines showed significant enhancement of root growth inhibition as compared to wild-type (**Figures 8B–D**). The root growth of overexpression and wild-type *Arabidopsis* lines was estimated by root length and fresh root weight. The statistical analysis showed that the root length and fresh weight of overexpression *Arabidopsis* lines were significantly inhibited as compared to wild-type *Arabidopsis*, as shown in **Figures 8E, F**. Therefore, we conclude that the root growth of transgenic *Arabidopsis* carrying the peanut *AhGRFi* gene is sensitive to NAA.

### 3.9 Expressions of auxin-responsive genes in *AhGRFi* overexpressing *Arabidopsis*

**Figure 9:**
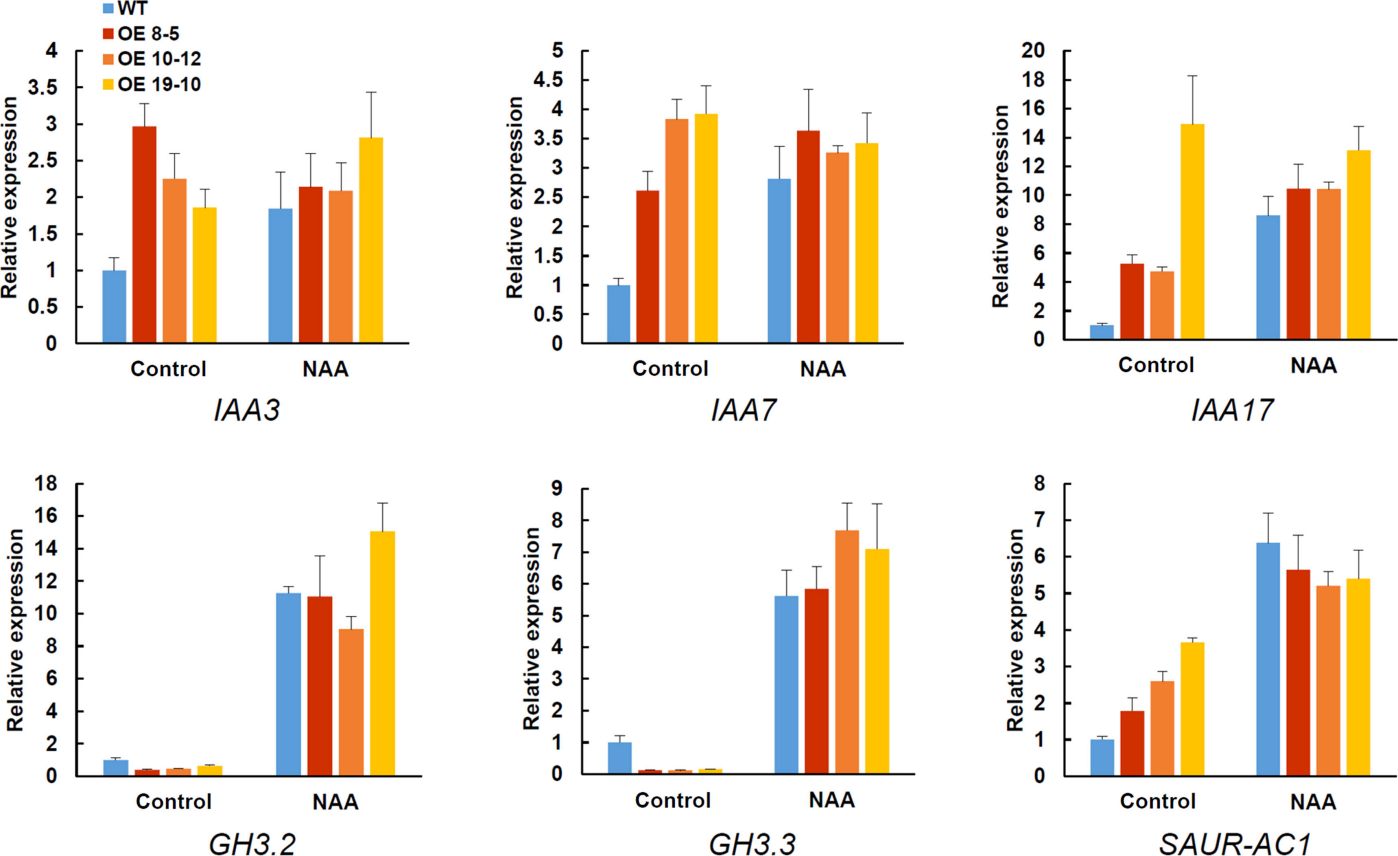
Real-time PCR analysis of auxin-responsive genes in transgenic *Arabidopsis* overexpressing *AhGRFi*. The relative expression values were normalized to the *Arabidopsis Actin2* gene. Error bars represent the means ± SE of three replications.

The effect of *AhGRFi* overexpression on auxin-responsive genes such as *IAA3*, *IAA7*, *IAA17*, *GH2.2*, *GH2.3*, and *SAUR-AC1* was further investigated by *q*RT-PCR. As shown in **Figure 9**, the expression of auxin-responsive genes such as *IAA3*, *IAA7*, *IAA17*, and *SAUR-AC1* was upregulated in *AhGRFi*-overexpressing *Arabidopsis*, while *GH3.2* and *GH3.3* were downregulated. Under NAA treatment, the expression levels of *IAA3*, *IAA7*, and *IAA17* were still relatively high, but the expression of *GH3.2* and *GH3.3* showed opposite trends of change, whereas the upregulation of *SAUR-AC1* expression is slightly reduced compared to the wild-type plant.

## 4 Discussion

The AhGRFi–YFP fusion protein transiently expressed in tobacco epidermal cells showed a yellow fluorescent signal distribution in the cytoplasm but not in the nucleus. 14-3-3 protein is a kind of widespread protein in eukaryotic cells called “scaffold protein,” because the protein monomer state usually has no biological function. Through the formation of homologous or heterologous dimers, 14-3-3 protein is involved in the physiological regulation of cellular processes, including phosphorylation and signal transduction processes, by binding to phosphorylated proteins (Boer et al., 2013). There are two cases about the localization of 14-3-3 protein in cells: one is that the 14-3-3 protein is in the intracellular protein itself, and another is that the 14-3-3 protein binds to other proteins with the aid of the target protein to change the location of the information itself, which is involved in the regulation of complex biological processes (Paul et al., 2005). Bioinformatics structure prediction of peanut 14-3-3 proteins revealed that the protein has no signal peptide or transmembrane domain; thus, protein translation without modifications may be directly involved in the regulation of cell activities. The fusion protein of *Arabidopsis* 14-3-3 and fluorescent protein, expression, and localization of 14-3-3 proteins in cells were identified in tobacco protoplasts and onion cells, and it was found that different members of the 14-3-3 family of *A. thaliana* and their locations are different, which are located in the cytoplasm and nucleus and presumably play a different role in cell signal transduction processes (Sehnke et al., 2002). The rice 14-3-3 proteins were localized in the nucleus (Chen et al., 2006). Therefore, different members of the family of 14-3-3 proteins in the same species have different functions; the 14-3-3 proteins of different species have high homology, but their functions are also likely to differ very far. The positioning mechanism of 14-3-3 protein, in the aspect of plant hormone signal transduction and adversity stress response, is not only able to regulate the activity of the target protein and be functional but is also indirectly involved in gene transcriptional regulation (Himmelbach et al., 2003; Ishida et al., 2004; Wang et al., 2011).

Previous studies have found that 14-3-3 protein binds to target proteins via phosphorylation recognition sequence specificity, to participate in a complex regulatory network of cell biology. In plants, *14-3-3* genes are involved in plant growth and development processes including biotic and abiotic stress responses, plant hormone signal transduction, metabolism process regulation, cell growth and division, etc. (Yaffe and Elia, 2001). For instance, in this study, expression analysis showed very high expressions of *AhGRFc* and *AhGRFd* under aluminum stress. The expression pattern of the cotton *14-3-3* gene found that the cotton *14-3-3* proteins are involved in cotton root growth and drought stress response. In the case of corn, the *14-3-3* gene expression of corn *ZmGF14-6* as well as tomato and rape *14-3-3* gene expression is affected by stress (Sun et al., 2014). Gene expression pattern showed that *AhGRF*s had the highest expression level in peanut seeds and seedling root; in different developmental stages of peanut seeds, the expression of *AhGRF*s was relatively stable, and the expression level at 20 d and 30 d after pegging stage was slightly lower than in the later stages (**Figure 5**). The reason behind this is that 14-3-3 protein is involved in the process of plant hormone signal transduction, such as the ABA and GA signaling pathways, which can affect the growth and development of plants (Sehnke et al., 2002). It is reported that overexpression of the *Ta14-3-3* gene in *Arabidopsis* can inhibit root and seedling growth (Li et al., 2013). In a study of wild soybean GsGF14o, overexpression causes changes in *Arabidopsis* morphology, including small pores and short hair, under drought stress in transgenic *A. thaliana*, in addition to slower root growth (Sun et al., 2014).

Plant 14-3-3 proteins have been found to be associated with plant stress response (Hajibarat et al., 2022; Huang et al., 2022) and involved in a network of interactions that finely regulate signaling and homeostasis of multiple hormones such as abscisic acid (ABA) (Schoonheim et al., 2009), gibberellins (GAs) (Ito et al., 2014), ethylene (Catalá et al., 2014), and auxin (Takahashi et al., 2012; Camoni et al., 2018). In our previous studies, stress treatments such as drought, salt, high and low temperatures, and osmotic testing were applied to *AhGRFi*-overexpressing *Arabidopsis* lines, but we did not find significant phenotypic differences (data not shown). ABA, 1-aminocyclopropane-1-carboxylic acid (ACC), NAA, GA3, and paclobutrazol (PAC) treatments had been applied to transgenic plants, but except for NAA, there were no significant changes in other treatments (data not shown). The experimental results demonstrated that under NAA treatment, *AhGRFi* overexpression significantly inhibited root growth in *Arabidopsis* seedlings. Further analysis indicated that the expression of auxin-responsive genes in transgenic *Arabidopsis* behaved differently under NAA treatment. The early auxin-responsive genes *GH3.2* and *GH3.3* were downregulated under normal conditions but upregulated under treatment. *GH3* genes inactivate auxin to maintain the dynamic balance of auxin in plants (Lin et al., 2022), and upregulation of *GH3* genes enhances plant response to low concentrations of auxin (Chapman and Estelle, 2009). The combined effect of multiple genes may lead to the inhibition of root growth under exogenous NAA treatment, but it did not affect the growth of roots under normal culture conditions. The result suggests that this 14-3-3 protein in peanuts may be involved in the regulation of root growth by auxin signaling.

## Data availability statement

The datasets presented in this study can be found in online repositories. The names of the repository/repositories and accession number(s) can be found below: BioProject accession numbers: PRJNA243319, PRJNA773958, PRJNA687108, PRJNA638812, PRJNA555172, PRJNA525247, PRJNA511663, PRJNA517600, PRJNA503795, PRJNA498570, and PRJNA291488.

## Author contributions

YL and YH conceived this study and designed the experiment. ZZ, SG, SC, HL, JX, ZW, and YL performed the experiments and data analysis. SG, RM, and RD performed bioinformatics analysis. ZZ and SG wrote the manuscript. YL, SG, and YH completed the final revision. All authors contributed to the article and approved the submitted version.

## Supplementary material

Supplementary Figure 1 Genetic map of *AhGRF* gene family.

Supplementary Figure 2 Cloning of *AhGRFi*. M:DL2000 Marker, 1:cDNA of *AhGRFi*

Supplementary Figure 3 Detection of *AhGRFi* gene by leaf PCR of *Aradopsis* plants. M: DL2000 Marker, lane 1: vector control, lane 2: wild-type control, lanes 3-24: *AhGRFi* transgenic samples.

Supplementary Figure 4 qRT-PCR analysis of *AhGRFi* gene expression in *Arabidopsis* transgenic lines. The relative expression values were normalized to the *Arabidopsis Actin2* and *UBQ10* gene. Error bars represent the means ± SE of three replication.
