# Supplementary figures and images for: Overexpression of peanut (Arachis hypogaea L.) AhGRFi gene enhanced root growth inhibition under exogenous NAA treatment in Arabidopsis thaliana
Source: Front Plant Sci. 2023 Jun 21;14:1184058. doi: 10.3389/fpls.2023.1184058 (PMC10321354; doi:10.3389/fpls.2023.1184058)

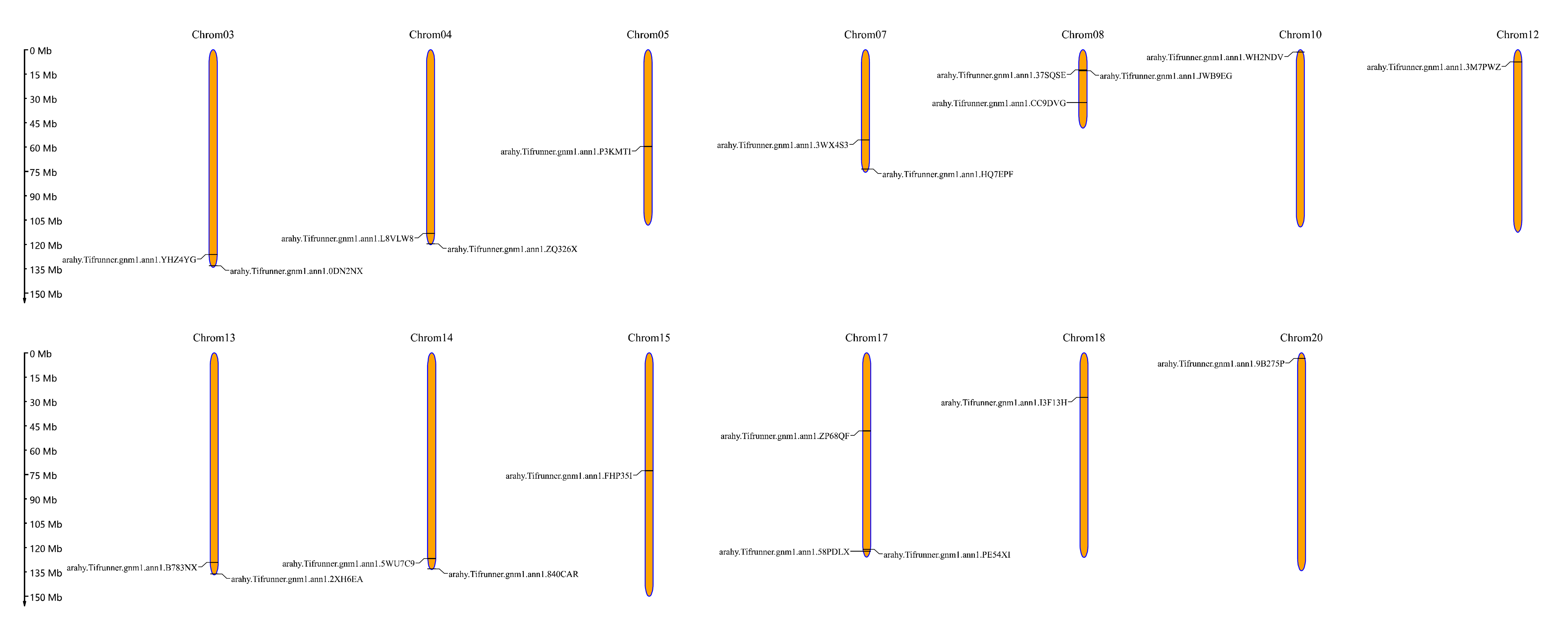

Supplement: Supplementary Figure 1 — Genetic map of AhGRF gene family. [file Image_1.jpeg]

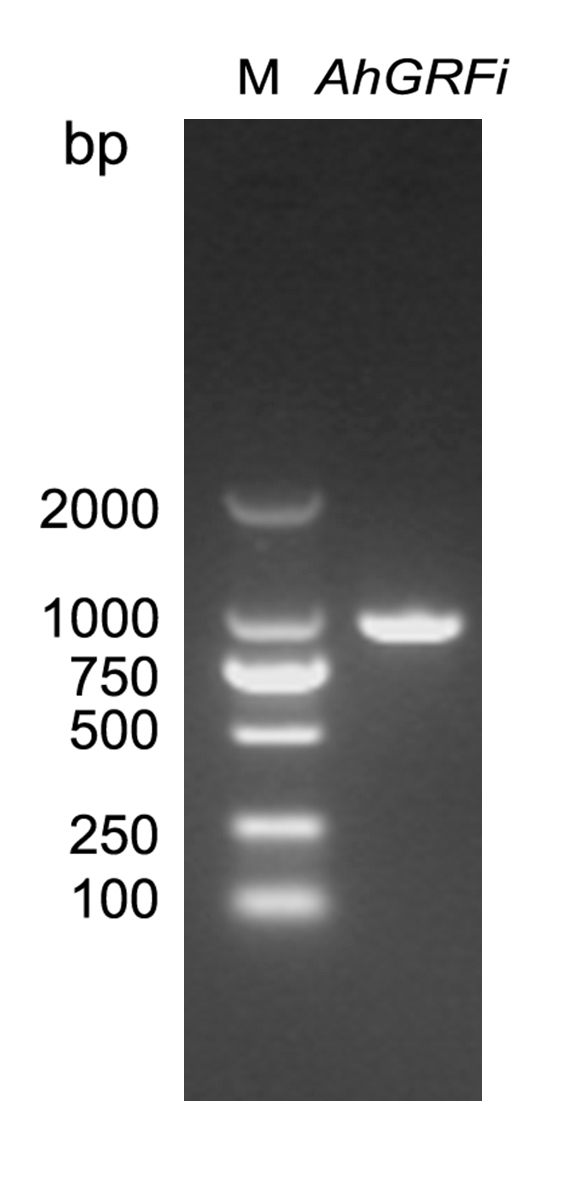

Supplement: Supplementary Figure 2 — Cloning of AhGRFi. M:DL2000 Marker, 1:cDNA of AhGRFi [file Image_2.jpeg]

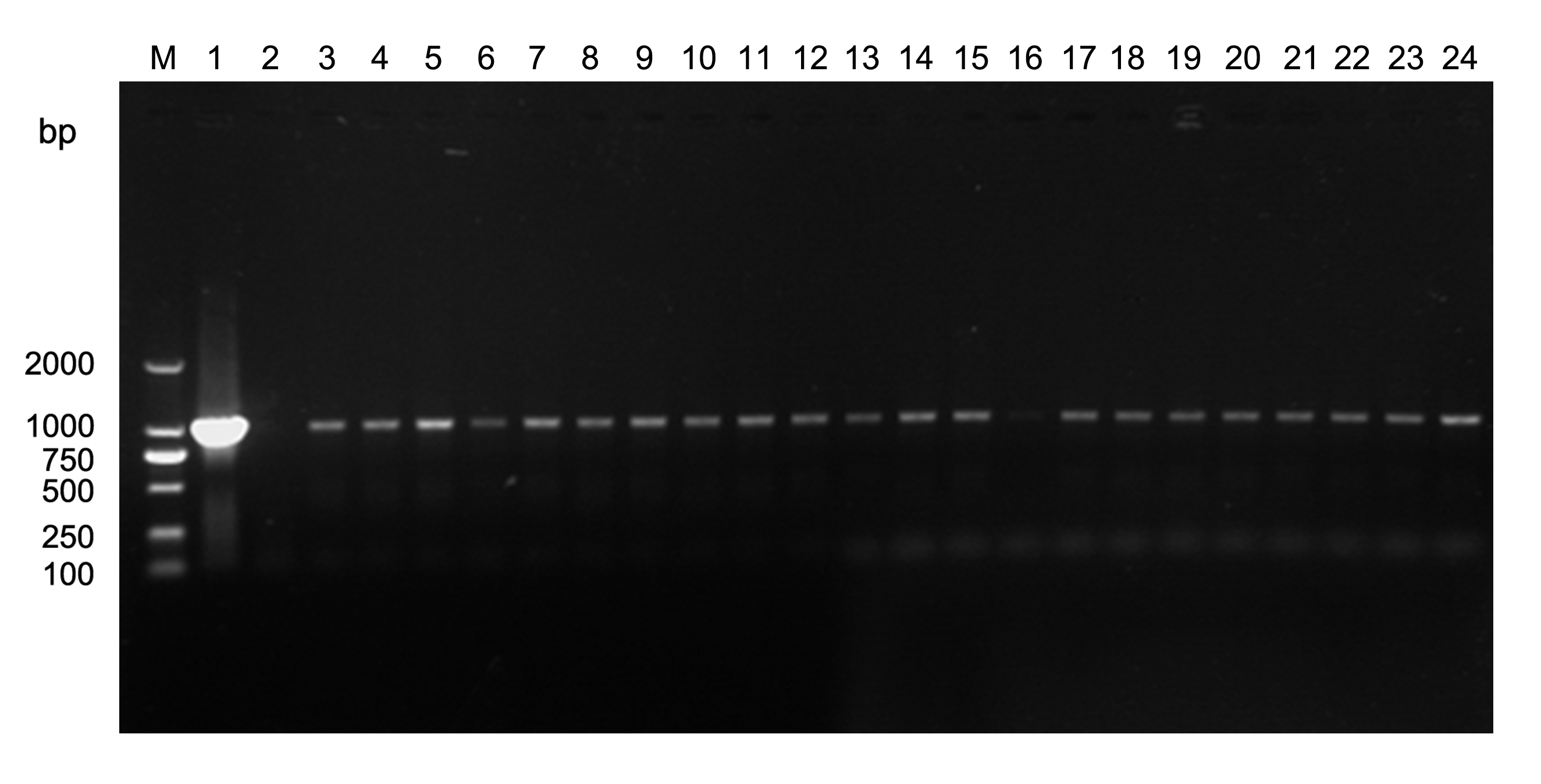

Supplement: Supplementary Figure 3 — Detection of AhGRFi gene by leaf PCR of Aradopsis plants. M: DL2000 Marker, lane 1: vector control, lane 2: wild-type control, lanes 3-24: AhGRFi transgenic samples. [file Image_3.jpeg]

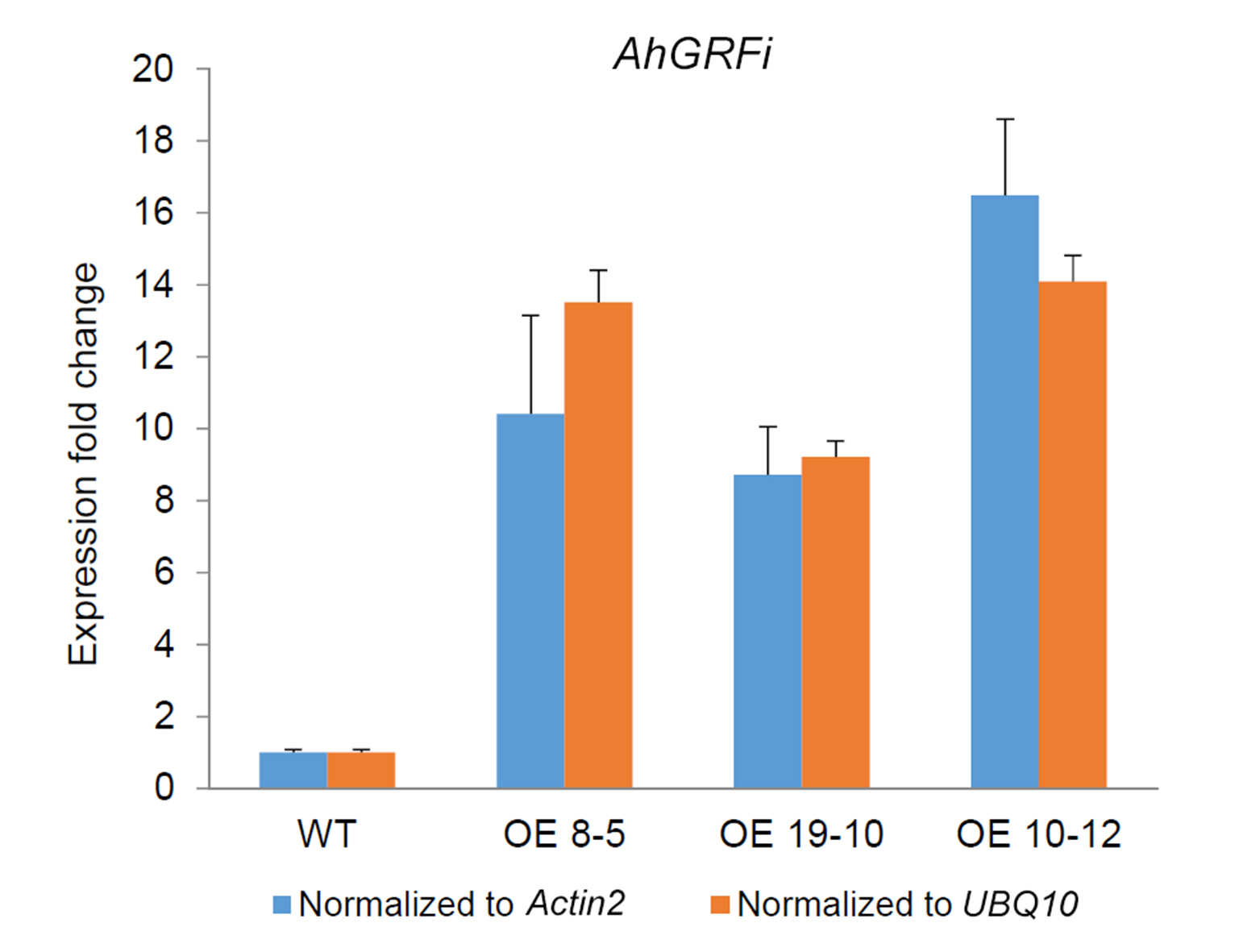

Supplement: Supplementary Figure 4 — qRT-PCR analysis of AhGRFi gene expression in Arabidopsis transgenic lines. The relative expression values were normalized to the Arabidopsis Actin2 and UBQ10 gene. Error bars represent the means ± SE of three replication. [file Image_4.jpeg]
