# Supplementary material for: Overexpression of peanut (Arachis hypogaea L.) AhGRFi gene enhanced root growth inhibition under exogenous NAA treatment in Arabidopsis thaliana
Source: Front Plant Sci. 2023 Jun 21;14:1184058. doi: 10.3389/fpls.2023.1184058 (PMC10321354; doi:10.3389/fpls.2023.1184058)
Supplement: Supplementary file 5 [file Table_1.docx]

Table S1. Primer sequences

| **Primers** | **Sequences (5’-3’)** |
| --- | --- |
| **For *AhGRFi* cDNA cloning** | |
| AhGRFi-F | AGAAACTCGCGAACTGAAAAA |
| AhGRFi -R | ATTGAAACATTGGAAACCACA |
| **For Subcellular Localization construction (restriction site underlined)** | |
| pA7-GRFi-F | GCGTCGACATGGCGGAATCATCTCG |
| pA7-GRFi-R | TCCCCCGGGCTGCTGCCCCTCGCCTGGTTGT |
| **For overexpressing construction (restriction site underlined)** | |
| p121-GRFi-F | TCCCCCGGGAGAAACTCGCGAACTGAAAAA |
| p121-GRFi-R | CGAGCTCATTGAAACATTGGAAACC |
| **For real-time PCR analysis of *AhGRFi expression*** | |
| QGRFi-F | GATCTGTGATGGCATTTTGA |
| QGRFi-R | CCAGCCTTATCGGGTGAGTA |
| Ah18SF | ATTCCTAGTAAGCGCGAGTCATCAG |
| Ah18SR | CAATGATCCTTCCGCAGGTTCAC |
| **For yeast hybrid construction (restriction site underlined)** | |
| ADGRFi-F | TCCCCCGGGATGGCGGAATCATCTCGCGAGGAGA |
| ADGRFi-R | CGGGATCCCTACTGCTGCCCCTCGC |
| BDGRFi-F | GCGTCGACATGGCGGAATCATCTCG |
| BDGRFi-R | TCCCCCGGGCTGCTGCCCCTCGCCTGGTTGT |
| **For real-time PCR analysis of NAA-related genes in *AhGRFi* transgenic *Arabidopsis*** | |
| IAA3-F | ACTGAAACATCCCCTCCTCG |
| IAA3-R | CCTTGACCCTCATGCTCAGA |
| IAA7-F | GTGGGAAGCAAGAGAGGCTT |
| IAA7-R | TGTGCTTTAGCAGGAGGCTT |
| IAA17-F | AAGATCCAGCCAAACCTCCG |
| IAA17-R | CGGTGCTCCGTCCATTGATA |
| GH3.2-F | TAGCGGTGGATTACCGATGG |
| GH3.2-R | TGCTTCTGCTGCTCCATCTC |
| GH3.3-F | TGAGTACTATAGCGGCGGGT |
| GH3.3-R | GCTAGCTCCACAAGTTCGGA |
| SAUR-AC1-F | CCGAAGAAGGACCATGTGTGGCA |
| SAUR-AC1-R | TGTGTCTGAGTGCTCCATGGCT |
| Actin2-F | AGATGCCCAGAAGTCTTGTTCC |
| Actin2-R | TTTGCTCATACGGTCAGCGATA |
| UBQ10-F | TACTTTGGCGGATTACAACATC |
| UBQ10-R | GAATACCTCCTTGTCCTGGATCT |
